# Role of three-dimensional printing and laser scanning in aesthetic restoration of Parry Romberg’s disease using de-epithelialized anterolateral thigh flap: a case report

**DOI:** 10.1093/jscr/rjae469

**Published:** 2024-08-16

**Authors:** Anupama Kumari, Veena Singh, Ansarul Haq, Sarsij Sharma, Niraj Bhalara

**Affiliations:** Department of Burns and Plastic Surgery, All India Institute of Medical Sciences Phulwarisharif, Patna 801507, India; Department of Burns and Plastic Surgery, All India Institute of Medical Sciences Phulwarisharif, Patna 801507, India; Department of Burns and Plastic Surgery, All India Institute of Medical Sciences Phulwarisharif, Patna 801507, India; Department of Burns and Plastic Surgery, All India Institute of Medical Sciences Phulwarisharif, Patna 801507, India; Department of Burns and Plastic Surgery, All India Institute of Medical Sciences Phulwarisharif, Patna 801507, India

**Keywords:** Parry Romberg syndrome, ALT flap, 3 D printing

## Abstract

Parry Romberg syndrome also known as progressive hemifacial atrophy is an uncommon degenerative condition, characterized by unilateral, slow, and progressive atrophy of face. Patient presents with loss of facial symmetry and neurological manifestations. After the degenerative process settles, reconstructive surgeries are performed to address facial asymmetry. For accurate assessment of volume deficit, laser scanning and three- dimensional printing can be used which offers the advantage of precise surgical planning and good aesthetic outcome. We present a case of soft tissue reconstruction in Parry Romberg syndrome with anterolateral thigh flap with use of three- dimensional laser scanning.

## Introduction

Parry Romberg syndrome also known as progressive hemifacial atrophy (PHA) is an acquired, typically unilateral distortion of face, due to atrophy of skin, soft tissues and muscles with occasional bony involvement. [1] It was first described by Parry in 1825 [2] and Romberg in 1846 [3]. Disease manifestation starts in first or early second decade of life affecting women slightly more than men [4]. True incidence of disease is unknown due to its rare presentation, overlapping features with linear scleroderma and lack of standardized diagnostic criteria [5]. Exact etio pathogenesis is yet not defined, however, various theories like auto-immunity, viral infections, trauma and inflammation of brain has been proposed [6]. Patients usually require aesthetic restoration of underlying soft tissue and bony defects. Reconstructive options include autologous fat grafting and tissue transfer, depending on severity of disease, degree of deformity, patient compatibility and surgeons’ preference.

## Case report

**Figure 1:**
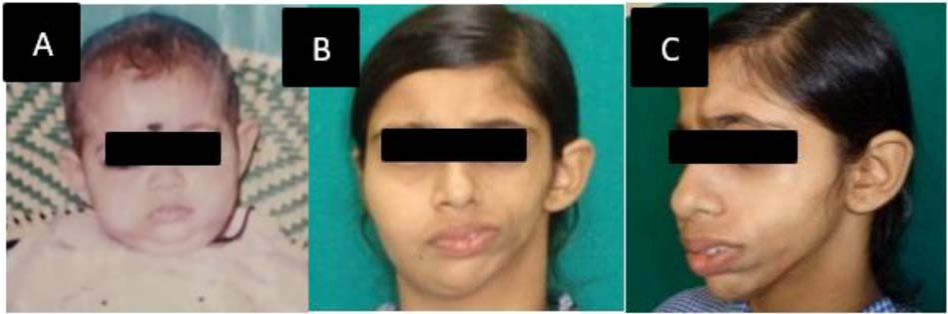
(A) Patient at 6 months of age without any deformity. (B, C) At presentation front and left oblique facial profile.

**Figure 2:**
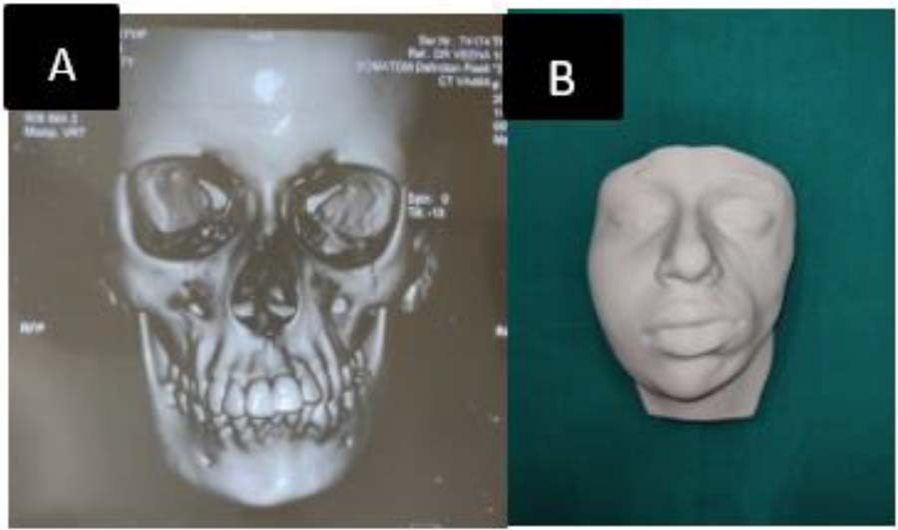
(A) Preoperative CT showing shortened left hemimandible. (B) 3 D model.

A 17-years-female presented to our tertiary center concerned for her left sided facial deformity. At birth no such deformity was noticed by the parents (Fig. 1A), However, -gradual change in contour and sunken in appearance of left side of face was appreciated at age of 2 years. Parents also gave history of accidental fall of an iron box over left side of her face and history of epileptic episodes during sleep, for 5 years of age. Physical examination revealed marked facial asymmetry with hypoplasia of left side of face and deviation of nose, lips, and chin to left side (Fig. 1 B and C). Facial nerve examination was normal. Patient was investigated and parents were counselled regarding the nature of disease, treatment options and outcomes. Three -dimensional computed tomography scan (3 D- CT) revealed, shortened ramus of left hemimadible with subcutaneous fat atrophy of left anterior maxillary, zygomatic, left parotid, buccal, and masseter space (Fig. 2 A). Laser scanning and 3 D printing was done for accurate defect analysis and planning of volume restoration (Fig. 2 B). The atrophic area was ~10 cm × 5 cm with a volume discrepancy of 299 ml between the left and right face. A free anterolateral thigh (ALT) flap was planned and counselling was done for additional corrective surgeries if required.

### Intraoperative details

**Figure 3:**
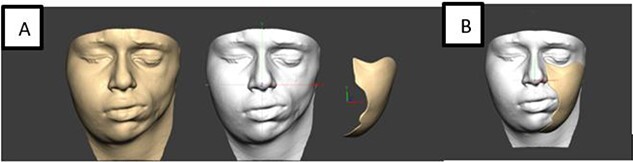
(A) Laser scanning for defect assessment. (B) Template.

**Figure 4:**
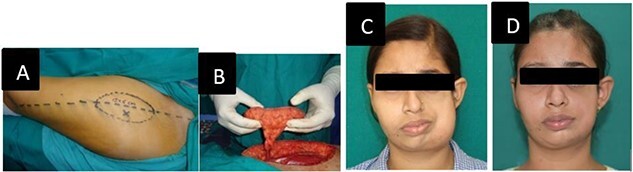
(A) Free anterolateral thigh flap preoperative marking. (B) Flap after harvest and ready for transfer. (C) After free ALT flap. (D) After flap debulking surgery.

Marking of atrophied site of face was done and template of defect was made using 3 D laser scanning (Fig. 3). For perforator mapping, a line drawn from superolateral aspect of patella to the anterosuperior iliac spine on the ipsilateral thigh of affected site (Fig. 4 A). A 3-cm-radius circle was drawn, centered on midpoint of this line and with use of a handheld Doppler probe; perforators inside the circle were identified followed by marking of the desired flap. A 12 cm × 6 cm flap based on single musculocutaneous perforator was harvested (Fig. 4 B). The flap was planned larger than estimated dimensions, due to the retractile properties of skin and to compensate for contraction. It was done so because, if a flap is stretched after harvest to fit the defect, it may reduce its viability leading to necrosis [7]. Preparation of the facial pocket was done and skin and subcutaneous tissue was elevated towards the nasolabial fold along with dissection of recipient vessels (facial artery and facial vein). The harvested flap was de-epithelialized and secured to periosteum of zygoma and infra-orbital rim and mandible. Primary closure of donor site was done. Flap settled well without any complications. Post-operatively no seroma, wound dehiscence or flap necrosis was seen. Donor site healed well. A second stage surgery for contour restoration was done after 18 months in form of flap debulking. There was dramatic improvement in facial contour and symmetry which was satisfactory for the patient.

## Discussion

PHA results in marked facial asymmetry which leads to psychological upset [8]. The condition is self- limiting and progresses for years to decades before cessation [9]. Diagnosis is based on detailed history, clinical examination and certain specialized tests based on individual disease manifestation. Hemifacial atrophy following trauma is rare. In our case, asymmetry was noticed by parents following trauma. Surgical treatment is usually done after the cessation of atrophic process and options include autologous fat grafting, pedicled, or free flaps depending upon the defect. At times, more than one treatment modality or a combination may be required [10].

For moderate to severe fat atrophy associated with bony hypoplasia, free tissue transfer is usually warranted. Various free flaps have been discussed in literature including latissimus dorsi [11], radial forearm, scapular and parascapular and ALT flaps. Since its first description by Song *et al.,* ALT flap has been one of ideal donor site for soft tissue reconstruction as the flap bulk can mask underlying bony hypoplasia and the donor site closure is possible with inconspicuous scar. Literature review describes a large series of ALT flap for facial contour restoration in case of PRS with its various modifications. Recently, use of laser scanning and 3 D printing for good aesthetic outcome has also been illustrated. In our case 3D printing was used to achieve our reconstructive goal of normal fullness and contour. We did address mandibular hypoplasia by bulk of ALT. Recent advancement in computer-aided design and manufacturing techniques has made feasibility of custom-made artificial bones possible. While these procedures have specific advantages, their use is limited due to stability and strength of implants. Also, use of any artificial material is associated with the risks of damage and infection, along with expulsion of implant. Hence, Brawn *et al.,* has preferred autologous tissue reconstruction due to their long-term outcome [12]. In a recent case series of three patients by K.F. Abdullaev *et al*., defects resulting from PRS and hemifacial microsomia were addressed with ALT flap, good and satisfactory aesthetic results were obtained. In third patient, with 3-D visualization, detailed analysis of defect and proper preoperative planning good contour restoration was achieved after first surgery only [13].

## Conclusion

Facial contour restoration is a challenge for reconstructive surgeon in PRS. Proper planning, analysis of components of defect and discussion of various reconstructive options with patient is mandatory. 3 D printing reduces number of procedures required and results in good aesthetic outcome.

## Data Availability

Data sharing is not applicable to this article as no datasets were generated or analysed during the current study.
